# Specialized Bacteroidetes dominate the Arctic Ocean during marine spring blooms

**DOI:** 10.3389/fmicb.2024.1481702

**Published:** 2024-11-05

**Authors:** Álvaro Redondo-Río, Christopher J. Mundy, Javier Tamames, Carlos Pedrós-Alió

**Affiliations:** ^1^Department of Systems Biology, Centro Nacional de Biotecnología, CSIC, Madrid, Spain; ^2^Centre for Earth Observation Science, Clayton H. Riddell Faculty of Environment, Earth, and Resources, University of Manitoba, Winnipeg, MB, Canada

**Keywords:** metagenomics, marine, Arctic, Bacteroidetes, cazymes, polysaccharides

## Abstract

A metagenomic time series from Arctic seawater was obtained from Dease Strait, to analyse the changes in bacterioplankton caused by the summer phytoplankton bloom. Bacterial clades specialized in the metabolism of polysaccharides, such as Bacteroidetes, became dominant along the bloom. These specialized taxa quickly displaced the microbial clades that dominate nutrient-poor waters during early spring, such as Archaea, Alpha-and Gammaproteobacteria. At the functional level, phyla Bacteroidetes, Planctomycetes, and Verrucomicrobia showed higher contents of polysaccharide-degradation functions. The Bacteroidetes community shifted toward species with higher polysaccharide-degrading capabilities, targeting algal polysaccharides in summer. Regarding transporters, Bacteroidetes dominated SusC-TonB transporters and had an exclusive family of glycoside-binding proteins (SusD). These proteins were used to identify polysaccharide-utilization loci that clustered transporters and polysaccharide-active enzymes, showing a higher level of specialization toward polysaccharide use. Altogether, these genomic features point to the genetic adaptations that promote the dominance of Bacteroidetes during phytoplankton blooms.

## 1 Introduction

Marine primary production accounts for about half the global photosynthetic carbon assimilation (Field et al., 1998). In recurrent phytoplankton blooms marine photosynthetic microorganisms flourish in coastal areas during spring or summer, driven by the rise in temperature, the increase in solar irradiation and the build-up of nutrients during winter.

During these blooms, lysis of microalgae by predators and viruses causes an increase in dissolved organic carbon (DOC), including diatom-synthesized polysaccharides. This DOC enrichment provides a suitable substrate for the proliferation of heterotrophic bacteria, which in turn remineralize the bulk of the fixed carbon. Some polysaccharides are resistant to bacterial hydrolysis and undergo sedimentation into the deep ocean, acting as a carbon sink. Therefore, these events are crucial for understanding global carbon cycling.

Phytoplankton blooms and the consequent bacterioplankton blooms have been thoroughly studied in temperate regions (Niu et al., 2011; Tada et al., 2011; Teeling et al., 2012, 2016) using single-cell labeling (BIC-FISH or CARD-FISH) and metagenomics. Since the main bacterial substrates during these events are polysaccharides, some studies have paid especial attention to the use that bacterioplankton make of these polymers (Teeling et al., 2012). Polysaccharides are synthesized by algae as cell wall constituents, carbon storage and extracellular mucilage. It has been estimated that laminarin alone, the primary storage polysaccharide in diatoms, represents 5–15 Gt of biomass produced annually (Alderkamp et al., 2007). However, most planktonic heteroglycans are still undescribed, with only some analyses revealing a wide variety of monomers and bonds, and a noticeable diversity among phytoplankton species (Gügi et al., 2015). Studies in polar regions have proven that water under ice receives enough solar radiation to sustain growth of ice-associated diatoms both in the Arctic (Lovejoy et al., 2007; Arrigo et al., 2012; Johnsen et al., 2018) and Antarctic (Torstensson et al., 2018). Planktonic diatoms are mainly responsible for spring–summer blooms in the Arctic Ocean, with a dominance of, among others, *Nitzschia frigida* and *Attheya ssp.*, belonging to clade Bacillariophyta, although dinoflagellates (Dinophyceae), *Phaeocystis* (Haptophyceae) and picoeucaryotes are also important in this environment (Pedrós-Alió et al., 2015).

Bacterial communities in both polar regions have been studied through 16S-rRNA analyses in the context of algal blooms (Alonso-Sáez et al., 2008; Comeau et al., 2011; Ghiglione et al., 2012), but only recently has metagenomics been applied to Arctic samples (Cao et al., 2020; Rego et al., 2021). Despite the existing research, a metagenomic time series covering a phytoplankton bloom is still lacking, and so does a closer examination of polysaccharide utilization in the whole bacterial community. Marine bacterial communities in polar regions are dominated by Alphaproteobacteria, Gammaproteobacteria and Bacteroidetes, with an important contribution of Actinobacteria, Deltaproteobacteria and Archaea (Cao et al., 2020). The degradation of marine polysaccharides by bacterioplankton requires a high metabolic specialization, including an appropriate repertoire of enzymes as well as an efficient system to take up the degradation products. Within bacterioplankton, only a few clades have developed the required capacity to efficiently degrade these polymers. Bacteroidetes is a bacterial phylum specialized in the degradation of polysaccharides (Fernández-Gómez et al., 2013). Thus, they are a dominant clade in different environments, from soil (Larsbrink and McKee, 2020) to the animal gut (Magne et al., 2020). In marine environments, Bacteroidetes are more abundant in both polar regions compared to temperate regions (Pedrós-Alió et al., 2015) for reasons possibly related to substrate availability. The metabolic specialization of this phylum is given by a genetic repertoire unique to them. Bacteroidetes are known for having a set of carbohydrate-active enzymes (cazymes) that they can secrete to degrade complex polymers into simpler, manageable oligomers, which they then take up through specialized transporters and binding proteins. Once in the periplasm, the oligomers can be efficiently degraded with no risk of other bacteria scavenging on the sugars. In Bacteroidetes species, cazymes and transporters have been found to be clustered in regulated loci known as polysaccharide utilization loci (PULs) (Martens et al., 2009). This type of locus was first described in the commensal species *Bacteroides thetaiotaomicron* (Reeves et al., 1997).

As mentioned, the Arctic Ocean experiences a bloom of phytoplankton in early summer that constitutes the main primary production event, accompanied by a profusion of polysaccharides. First, the algae growing attached to ice synthesize large amounts of extracellular polysaccharides that are disposed into the water column as the ice melts. And second, the blooming algae themselves produce storage and cell wall polysaccharides that are released because of predation. The composition of the bacterial assemblage changes dramatically during the bloom. Despite previous studies, a community-wide description of the polysaccharide degradation potential of Arctic bacteria is lacking, especially in the context of algal blooms. As most studies have focused on taxonomic descriptions, we still lack functional analyses establishing a connection between enzymatic functions and substrate availability. In the present work the objective was to describe the bacterial community dynamics using a time-series of metagenomes, and to determine whether the sets of polysaccharide-degrading enzymes of each bacterial group could, at least partially, explain the observed succession of taxa. Metagenomic data contains enough information to describe how different enzymatic functions are distributed among the taxonomic groups, together with their abundance and organization in the genome.

## 2 Methods

### 2.1 Sampling

Samples were collected at Dease Strait, Nunavut, Canada (69.03°N, 105.33°W). Seawater was sampled at 2.5 m deep during spring and summer of 2014 as part of the Ice Covered Ecosystem-CAMbridge Bay Process Study (ICE-CAMPS). Sampling was performed through holes in the ice from 7 March to 24 June. Ice melting started on 8 June, and ice break-up occurred on 19 July. The sampling on 30 July was performed from a boat in ice-free waters.

### 2.2 DNA extraction and high-throughput sequencing

Biomass was collected by filtering approximately 10 L of seawater through series of 20, 3, and 0.22 μm filters. The 0.22 μm filters were kept at −80°C until DNA extraction was performed. Total DNA from filter pieces was extracted using the phenol/chloroform protocol as described elsewhere (Massana et al., 2004). After thawing, the frozen filters were fragmented into small pieces and then placed into sterile cryovials. Lysis buffer and lysozyme were added, resulting in a final lysozyme concentration of 1 mg/mL, followed by a 45-min incubation at 37°C. Subsequently, sodium dodecyl sulfate (10%) and Proteinase K (0.2 mg/mL) were incorporated into the mixture, and this blend was incubated at 55°C for 1 h. The lysate underwent two rounds of mixing with an equal volume of phenol/chloroform/isoamyl alcohol (25:24:1, pH 8) and centrifugation at 12,000 × rpm for 10 min. To eliminate any residual phenol, a second extraction was performed using chloroform/IAA (24:1). The aqueous phase, containing the DNA, was then concentrated, and purified using Amicon Ultra Centrifugal filters from Millipore. Nanodrop spectrophotometer (NanoDrop 1000 Thermo Fisher) and a Qubit fluorimeter (Thermo Fisher) were used to assess DNA yield and integrity. Finally, the DNA extracts were kept at −80°C until sequencing.

Extracted DNA was sequenced at CNAG^1^ with an Illumina HiSeq2000 sequencing platform with a TruSeq paired-end cluster kit, v3. Samples were sequenced in two different batches, the second batch including samples dated May 10, June 1, June 15, and July 30. Details of the sequencing yield are included in Supplementary Table S1.

### 2.3 Bioinformatic analysis

The metagenomic analysis was carried out with pipeline SqueezeMeta v1.3 (Tamames and Puente-Sánchez, 2019) and results were later processed with the R package SQMtools (Puente-Sánchez et al., 2020). This pipeline performed all the necessary steps of metagenomic analysis from raw reads to binning. The pipeline was run in merged mode, which assembled reads from each sample individually using Megahit. Then, the contigs from all samples were combined, de-duplicated and re-assembled. The pipeline then inferred ORFs with Prodigal. Taxonomic assignment was done with Diamond homology searches against the NCBI nr database. Functional annotation used a combination of Diamond and HMMer homology searches against PFAM, KEGG and COG databases. Finally, coverage was calculated mapping the reads to the assembly with Bowtie read mapper. Further details on the functioning of the pipeline can be found in Github: https://github.com/jtamames/SqueezeMeta. The results of the assembly are detailed in Supplementary Tables S1, S2. Taxonomic composition was measured in percentages, so changes in these values do not represent actual growth or disappearance of the clades, but just dominance against the others. MDS analysis was performed with the *cmdstats* function in the *stats* R package using chi-squared distances calculated with the *vegdist* function from package *vegan*.

### 2.4 Function copy-number calculation

Copy-number was chosen to estimate functional content of bacterial clades. It was calculated for taxonomic subsets of the dataset, e.g., considering only sequences annotated as Bacteroidetes. For each of these subsets, copy number was calculated as the ratio between the summed coverage of all genes annotated with a given function and the median coverage of 11 Universal Single-Copy Genes (USiCGs, Supplementary Table S3). This transformation normalizes the coverage of a given function relative to the coverage of a set of genes with one single copy expected in each genome. In this way, the obtained copy numbers represented the average number of genes with a given function each clade has.

### 2.5 Functional annotation of ORFs

Sequences of all known cazymes have been collected in the CAZy database (Lombard et al., 2014). These enzymes are grouped in five classes, plus an additional class of non-catalytic carbohydrate-binding modules (CBM). The catalytic classes include glycosyl hydrolases (GH), glycosyl transferases (GT), polysaccharide lyases (PL), carbohydrate esterases (CE) and other accessory redox enzymes (AA).

Functional annotations for the specialized databases CAZy [v.07312020 (Cantarel et al., 2009)] and TIGRfam [v4.0 (Li et al., 2021)] were added to the PFAM, KEGG and COG annotations that were automatically carried out by SqueezeMeta. Annotations for CAZy were performed with Diamond v2.0.8.146, while annotations against TIGRfam were obtained with HMMer v3.1b2.

As the annotation of enzymatic function by homology is often ambiguous, we developed a consensus annotation (Bennke et al., 2016; Teeling et al., 2016), combining CAZy, PFAM, KEGG, and COG annotations to reduce false-positives. To establish an equivalence between PFAM, KEGG, and COG identifiers and CAZy families, all sequences in CAZy were annotated in these three databases. The retrieved annotations that were related directly with carbohydrates were selected, removing other more generic domains such as cofactor-binding sites. We only kept cazymes when at least one of the extra annotations (PFAM, KEGG, COG) was carbohydrate related. This removed spurious cazyme annotations in which a gene resembled a cazyme but was more similar to another protein not included in CAZy, which could only be detected by annotating against broad databases such as PFAM, KEGG, and COG. To test this approach, nine reference genomes were retrieved from PATRIC (Davis et al., 2020). The selected genomes belonged to marine bacterial species that had their curated cazyme annotation published in CAZy (Supplementary Table S4). The nine genomes were automatically annotated using the same pipeline used for the metagenomes, and then the CAZy annotation was refined using the proposed consensus approach. The results of this consensus annotation improved overall accuracy with a minimum reduction in recall (Table 1).

**Table 1:** Annotation of different cazyme groups in reference genomes.

	GH			GT			PL			CE		
	*N*	Rec (%)	Acc (%)	*N*	Rec (%)	Acc (%)	*N*	Rec (%)	Acc (%)	*N*	Rec (%)	Acc (%)
Ref.	458	–	–	365	–	–	40	–	–	30	–	–
Auto.	1,140	97	39	1,181	100	31	60	93	62	135	97	21
Filter	481	94	89	439	99	82	35	80	91	61	97	48

Rows show the reference curated annotations (Ref.), the automatically retrieved annotations without any filter (Auto.), and those obtained after applying the consensus filter. Columns show the total number of annotations (N) and the recall (Rec) and accuracy (Acc) percentages.

### 2.6 PUL identification

Polysaccharide Utilization Loci (PULs) were predicted with the criteria used to create the database PULDB (Terrapon et al., 2015). A PUL is described as a locus where a *susC-susD* gene pair is present and it is clustered with at least three cazymes. This gene pair comprises a TonB-dependent transporter (*susC*) and a polysaccharide-binding protein (*susD*). Genes were considered to be clustered if they were contiguous in the same direction and less than 102 bp apart, except for cazymes, that could exceed this limit. *SusC* genes were retrieved by looking for associated annotations PF00593 [TonB-dependent receptor], PF07715 [TonB-dependent receptor plug domain] and PF13715 [Carboxy-pepD domain] from the PFAM database, and annotation TIGR04056 [SusC/RagA Family] from TIGRfam; *susD* genes were retrieved with domains PF07980 [SusD Family], PF12741 [SusD and RagB], PF12771[Starch-binding SusD-like] and PF14322 [Starch-binding SusD-like N-terminal] from PFAM (Martens et al., 2009).

Contigs that had a *susC-susD* tandem and at least 3 cazymes were visualized using the DNA Features Viewer Python library (Zulko and Edinburgh Genome Foundry, 2021) to check the distance and direction criteria.

## 3 Results

### 3.1 Taxonomic diversity

**Figure 1:**
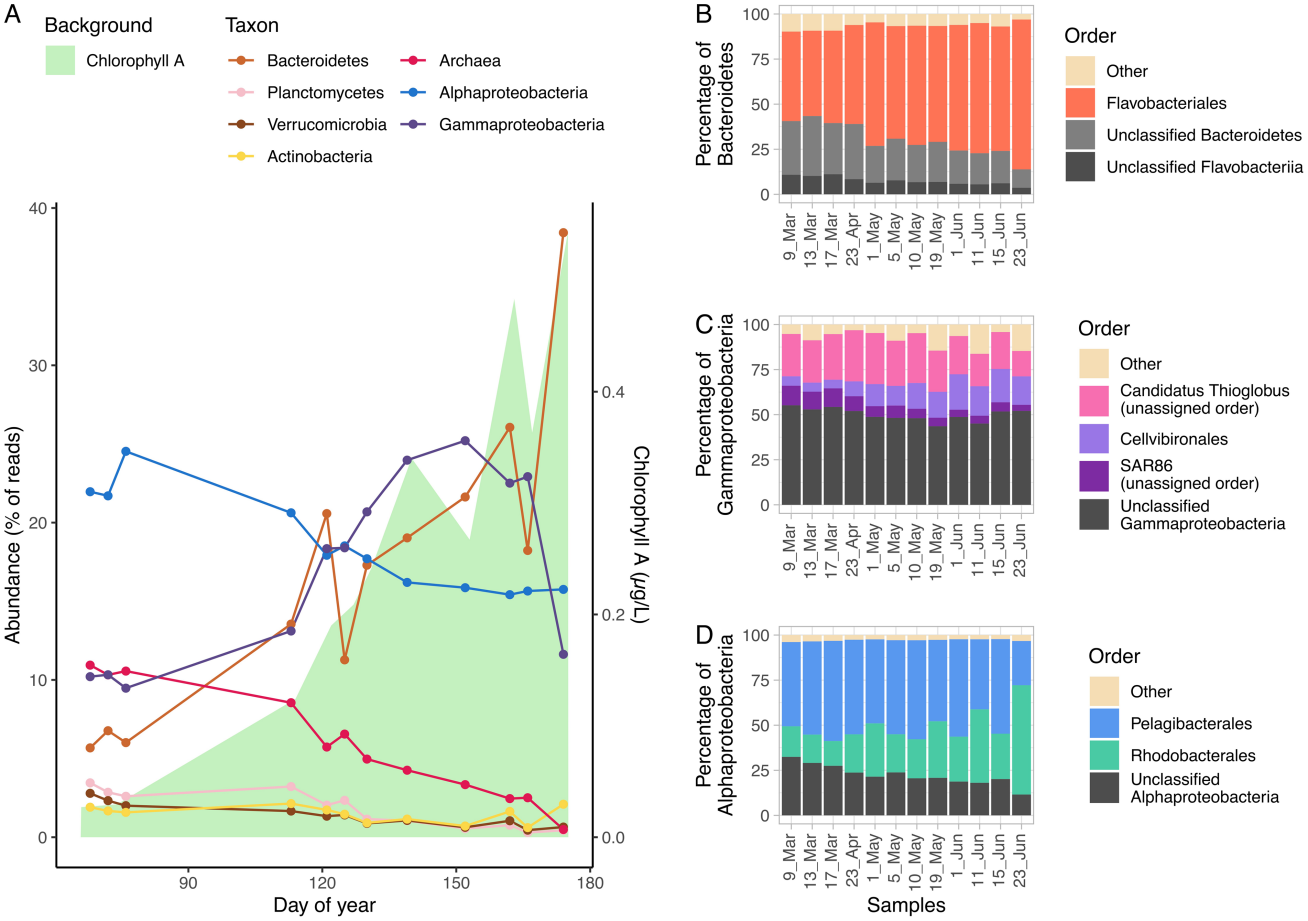
Percentage of reads of different taxonomic groups with time. The background area represents chlorophyll A (μg/L) in water **(A)**. Compositions of the Bacteroidetes phylum **(B)**, Gammaproteobacteria class **(C)**, and Alphaproteobacteria class **(D)** as percentage of reads across the time series with the most representative clades.

The time series could be divided in two periods. The first three samples corresponded to late winter, when ice was around 2 m thick and algae had not bloomed, although ice-associated diatom growth has been described in this period. The microalgae population during late winter was dominated by small flagellates. The remaining samples covered the increase in chlorophyll from early May to late June. As ice melting triggered the algal bloom, the chlorophyll peak occurred in July (Figure 1A).

Over 80% of the taxonomically classified reads belonged to phyla Proteobacteria (57.8%) and Bacteroidetes (24.4%). Within these phyla, three classes represented over 80% of the reads taxonomically classified at class level: Alphaproteobacteria (33.2%) and Gammaproteobacteria (28.7%) from the phylum Proteobacteria, and Flavobacteriia (21.6%), from the phylum Bacteroidetes. Alphaproteobacteria was the dominant clade during early spring (23% of reads), with the most abundant annotated orders being Pelagibacterales (50%) and Rhodobacterales (15%). Almost half of the Pelagibacterales reads were classified as *Candidatus* Pelagibacter. Gammaproteobacteria was the second most abundant class (10% of reads), with a dominance of *Candidatus* Thioglobus (almost 25% of the Gammaproteobacteria reads). Archaea were also abundant during this season, with over 10% of the reads. Bacteroidetes were at that time a minority, contributing around 6% of the reads, with a high proportion of Flavobacteriales (50% of the reads in the phylum). Other minority phyla were Actinobacteria, Verrucomicrobia and Planctomycetes, within the 1.5–3% range each (Figure 1A).

As the bloom progressed, Bacteroidetes steadily increased from slightly over 6% of the reads to over 17% in May and over 26% in June. Flavobacteriales were more dominant with time, representing over 70% of Bacteroidetes reads during May and June (Figure 1B). *Polaribacter* was the most abundant genus at that time, representing more than 20% of Flavobacteriales reads, despite comprising less than 4% of Flavobacteriales reads in late winter. Alphaproteobacteria, which were dominant in early spring, decreased to around 17% of the reads with a slight increase of Rhodobacterales, which grew from 15% of the class reads in early spring to over 20% in May and June, reaching a 60% maximum on June 23 (Figure 1D). Gammaproteobacteria increased rapidly from 10% to over 20% of the reads, reaching a 25% in early June and decreasing since then back to 11% at the end of the month. The most prominent groups in this class were *Candidatus* Thioglobus (20–30%), Cellvibrionales (5–15%) and the SAR86 cluster (5–10%). The *Cand*. Thioglobus clade (formerly included in the SUP05 clade) peaked at the beginning of the bloom, with almost 30% of the class reads, and decreased there on. Cellvibrionales increased together with the bloom, reaching a maximum of 20% in June and decreasing in the late bloom. The SAR86 clade was most abundant in early spring (10%) and decreased with the bloom, reaching a minimum of 3% in June (Figure 1C). Archaea decreased from 10% of the reads in early spring to around 2% by late June. The reads were mostly assigned to Nitrosopumilales (25–55%). Minority phyla (Actinobacteria, Planctomycetes, and Verrucomicrobia), showed a decreasing trend from around 3% in early spring to about 1.5% in late summer.

### 3.2 Functions in Arctic communities

**Figure 2:**
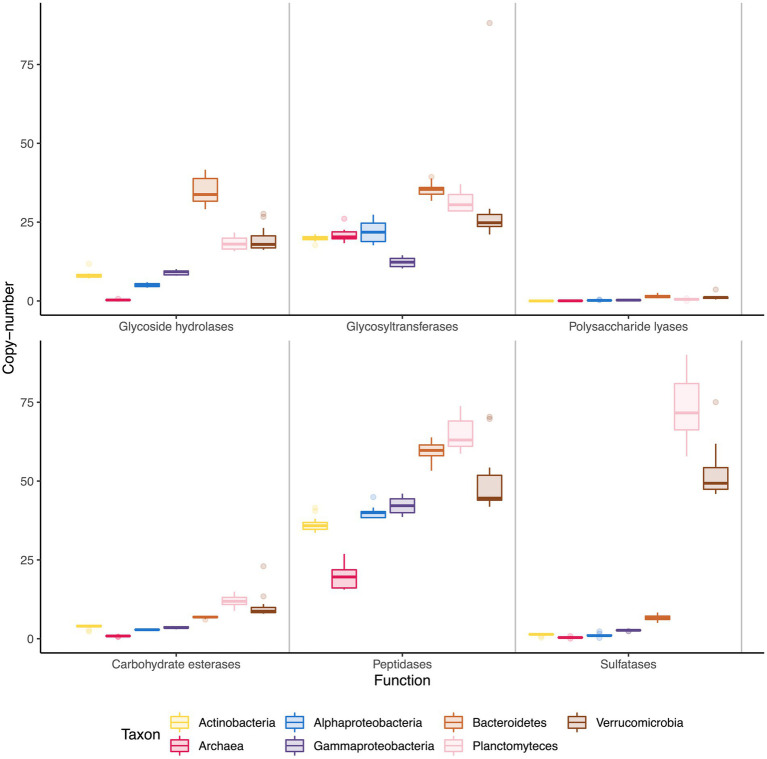
Copy number of relevant functions among taxonomic groups. Each box represents copy number variations across the time series, with the middle line representing the median value.

Copy number of the most relevant functions for the degradation of polysaccharides revealed that Bacteroidetes, Verrucomicrobia and Planctomycetes had a larger number of degradative enzymes than the rest of the clades (Figure 2), specifically, more catalytic cazymes (GH, GT, PL, and CE), sulfatases and peptidases than the other clades, though Bacteroidetes stood out as having more glycoside hydrolases. Archaea had a singular cazyme profile, having very low copy numbers for most degradative cazymes (GH, PL, CE) and sulfatases, and only having relatively high numbers of GT and peptidases. The main GT families annotated for Archaea were GT1, GT2, GT4, and GT66, related with glycoprotein biosynthesis, functions that are not related with polysaccharide use. Sulfatases were distributed unevenly, with extremely high numbers in the Planctomycetes and Verrucomicrobia phyla (73 and 52 copies) and markedly lower numbers in the remaining groups, with Bacteroidetes having at most 7 copies per genome.

### 3.3 GH profiles

To compare the enzymatic repertoires of different clades, the Chi-squared distance was calculated for the cazyme profiles of the considered taxa, using proportional copy-number data.

Broad unspecific families such as GH0 (unclassified), GH1, GH2, GH3 or families present in almost all groups (GH13 or GH65) were uninformative. However, most families in CAZy have a narrow functional annotation and are restricted to a few clades. Some of the GH families that were exclusive or mainly dominated by Bacteroidetes (Supplementary Figure S1) were GH30 (glucanase), GH92 (mannosidase), GH130 (mannose phosphorylase) or GH149 (glucose phosphorylase). Other GH families were only shared by Bacteroidetes with Planctomycetes or Verrucomicrobia, such as GH29 (fucosidase) and GH65 (phosphorylase). Planctomycetes and Verrucomicrobia also had some exclusive hydrolase families, most notably GH33, the only family with sialidase activity found in the dataset, and GH43, a family of arabinases and xylosidases. Proteobacteria shared two families of lysozymes, GH23 and GH73. Gammaproteobacteria dominated two other families, GH36 (*α*-galactosidase) and GH42 (*β*-galactosidase). Alphaproteobacteria had relatively less diversity of enzymes with no clear specialization (Supplementary Figure S1).

Transferases (GT) did not diverge so clearly among the different phyla (Supplementary Figure S2). GT profiles diverged the least of all the studied groups, with the lowest Chi-squared distance in GT family composition among the different taxa (Supplementary Table S5). Most groups shared the same families, namely GT2, GT4, and GT51, which are involved in peptidoglycan and glycolipid biosynthesis or post-translational modification of proteins, rather than in polysaccharide degradation. GT5 (glycogen synthase) was exclusive to Bacteroidetes, Planctomycetes and Verrucomicrobia and GT66 (glycoprotein biosynthesis), exclusive of Archaea. Proteobacteria shared GT41, involved in the post-translational modifications of adhesins.

Lyases (PL), a group of enzymes that specifically act on uronic acid residues, markedly varied among the groups (Supplementary Figure S3). However, most annotated polysaccharide lyases targeted alginate or pectin. Bacteroidetes and Gammaproteobacteria mostly relied on PL6, PL7 and PL17 for alginate lysis. Bacteroidetes had a unique family, PL8, targeting the mannuronic-rich regions in alginate. Planctomycetes and Verrucomicrobia, on the other hand, seemed to rely more on family PL14. Planctomycetes also had a great abundance of PL10, targeting pectin. Verrucomicrobia had less abundance of alginate-targeting lyases, and instead had PL2 and PL10 (pectate lyases) and PL12 (heparin lyase). Annotations in Actinobacteria, Alphaproteobacteria and Archaea were poor due to their low copy numbers.

Most bacteria shared the same families of esterases (CE, Supplementary Figure S4), devoted to removing O-and N-decorations from sugar monomers. Most targeted xylan (CE1 to CE7) and murein (CE9 and CE11). CE14 was common in the dataset, but it is poorly characterized in the CAZy database, with no experimental confirmation of the annotated chitobiose deacetylase activity. Verrucomicrobia and Planctomycetes were the only phyla containing CE15, which mainly represents glucuronic acid methylesterases (though no bacterial protein has been characterized). One must be weary of trusting these esterase annotations, as this enzyme group performed poorly in annotation (see Methods).

**Figure 3:**
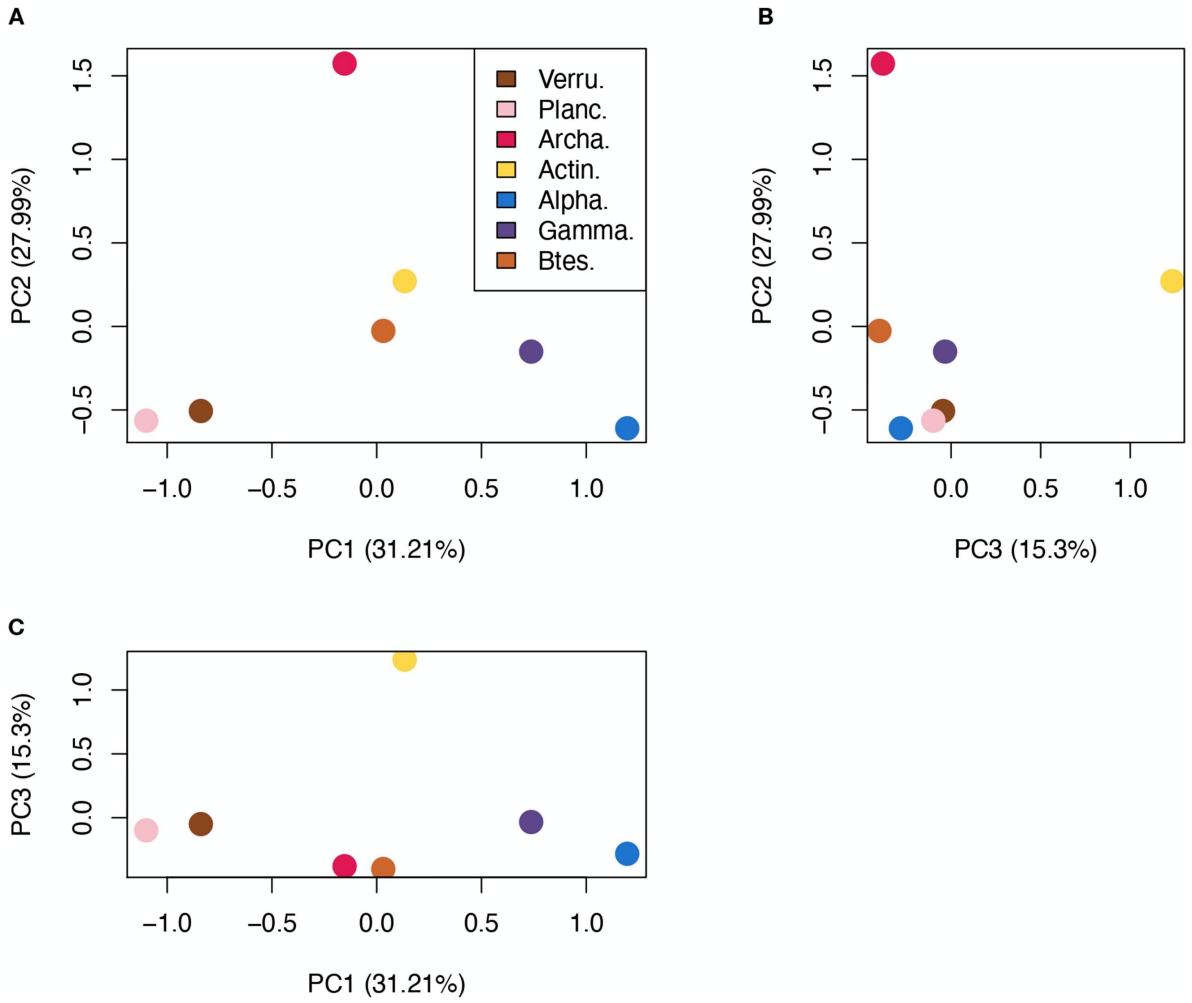
Metric MDS representation of GH profile χ2 distances. **(A)** PC1 vs. PC2, **(B)** PC2 vs. PC3, **(C)** PC1 vs. PC3. Each axis shows the explained percentage of variance. Total explained variance by the three components: 74.5%.

Differences in the cazyme profiles among taxonomic groups were assessed by calculating the chi-squared distance (Supplementary Table S5). This measure is commonly used for proportion data and is suitable to perform metric MDS. The selected clades diverged the most in GH and PL content, while GT and CE content generated lower distances, indicating more similarity. To better visualize the differences between GH profiles, which are the most abundant and most relevant enzymatic group, a metric MDS ordination was made (Figure 3). In this ordination, Archaea appear clearly apart from the rest of the taxa, showing their highly divergent GH content. Verrucomicrobia and Planctomycetes appeared in proximity, showing that these two phyla have very similar GH profiles, and clearly apart from both Proteobacteria classes, that seem to cluster at the opposite end of the first axis.

### 3.4 Cazymes in time

Changes in the bacterial community that accompanied the phytoplankton bloom could also be seen in the genetic content of the different clades at the enzyme family level. Bacteroidetes responded drastically to the bloom, with a sharp change in their cazyme content, while the rest of the clades did not show any remarkable change in their cazyme repertoire. The following section will thus focus on the changes at the genomic level in Bacteroidetes.

As spring came about, the copy number of cazymes increased in the Bacteroidetes. Glycosyl hydrolases increased from 30 copies per genome to over 40 in June. Such an increase was neither clearly observed in the rest of the clades nor for any other cazyme group. After seeing that the content of GH in Bacteroidetes increased during the bloom we checked whether this increase occurred in all GH families equally, or if some functions were more enriched than others during the bloom. The correlation between the copy-number of each GH family and chlorophyll was calculated with the Spearman correlation index. *p*-values for the correlation were corrected using the FDR approach, and only those families with a significant correlation (corrected *p*-value ≤ 0.01) are reported. Among the enzymes positively correlated with chlorophyll were those exclusive of Bacteroidetes (Table 2): GH30 (glucanase), GH92 (mannosidase), GH130 (mannose phosphorylase) and GH149 (glucan phosphorylase). Other highly correlated families were GH10 and GH120 (xylanases), GH17, GH81, and GH128 (laminarinases), GH148 (glucomannanase), GH86 (agarase), GH26, GH113, and GH125 (mannosidases) and some glucanase subfamilies in GH5 and GH13 (Supplementary Figure S2). Out of the 37 GH families and subfamilies that showed a positive correlation, 34 were directly related with polysaccharides, and not with disaccharides or glycoconjugates. Two families were related with the utilization of trehalose (GH37 and GH13_16) and one with sucrose (GH13_18), both anti-freezing and osmo-protectant disaccharides. Correlated families with the most significant *p*-values mostly targeted *β*-(1,3)-glucans (laminarin), β-(1,6)-glucans (laminarin branches) and mannans (Table 2).

**Table 2:** Top 10 GH families whose copy number in Bacteroidetes was positively correlated with chlorophyll with the highest significance.

GH family	Description	Corrected *p*-value	Substrate	Possible source
GH81	Endo-β-(1,3)-glucanase	0	Laminarin	Diatom storage
GH128	Endo-*β-*(1,3)-glucanase	0	Laminarin	Diatom storage
GH30_1	Endo-β-(1,6)-glucanase	0.000345	Laminarin	Diatom storage
GH149	β-(1,3)-glucan phosphorylase	0.000518	Laminarin	Diatom storage
GH17	β-(1,3)-glucanase	0.000518	Laminarin	Diatom storage
GH92	Exo-α-mannosidase	0	Mannan	Diatom cell wall
GH130	Mannose phosphorylase	0.000125	Mannan	Diatom cell wall
GH125	α-mannosidase	0.000125	Mannan	Diatom cell wall
GH13_31	α-(1,6)-glucosidase	0.000518	Starch	Dinoflagellate storage
GH37	Trehalase	0	Trehalose	Macroalgae

Corrected *p*-value corresponds to the Spearman’s correlation test after FDR correction.

Among the families that were negatively correlated with chlorophyll, many were unrelated with algal polysaccharides (Table 3): GH24 and GH25 (lysozymes), GH28 (pectin hydrolase), GH63, GH99, and GH1011 (glycoconjugates); but others represented enzymes that could act on polysaccharides: GH13_36 and GH15 (amylases), GH43 and GH51 (arabinases), GH74 (xyloglucanase), GH95 (fucosidase).

**Table 3:** Top 10 GH families whose copy number in Bacteroidetes was negatively correlated with chlorophyll with the highest significance.

GH family	Description	Corrected *p*-value	Substrate	Possible source
GH95	α-L-fucosidase	0	Fucoidan	Diatom mucilage
GH63	Glycoglycerate hydrolase	0	Glycoglycerate	Cyanobacteria
GH99	Glycoprotein mannosidase	0	Glycoproteins	Bacteria
GH101	Endo-α-N-acetylgalactosaminidase	0.00065	Glycoproteins	Bacteria
GH51	Hemicellulase	0.00081	Hemicellulose	Plants
GH25	Lysozyme	0.00042	Murein	Bacteria
GH24	Lysozyme	0.00076	Murein	Bacteria
GH43_4	Endo-α-(1,5)-L-arabinanase	0	Pectin	Plants
GH28	Polygalacturonase	0.00065	Pectin	Plants
GH74	Endo-glucanase specific to xyloglucan	0	Xyloglucan	Plants

Corrected *p*-value corresponds to the Spearman’s correlation test after FDR correction.

### 3.5 PULs in Bacteroidetes

**Figure 4:**
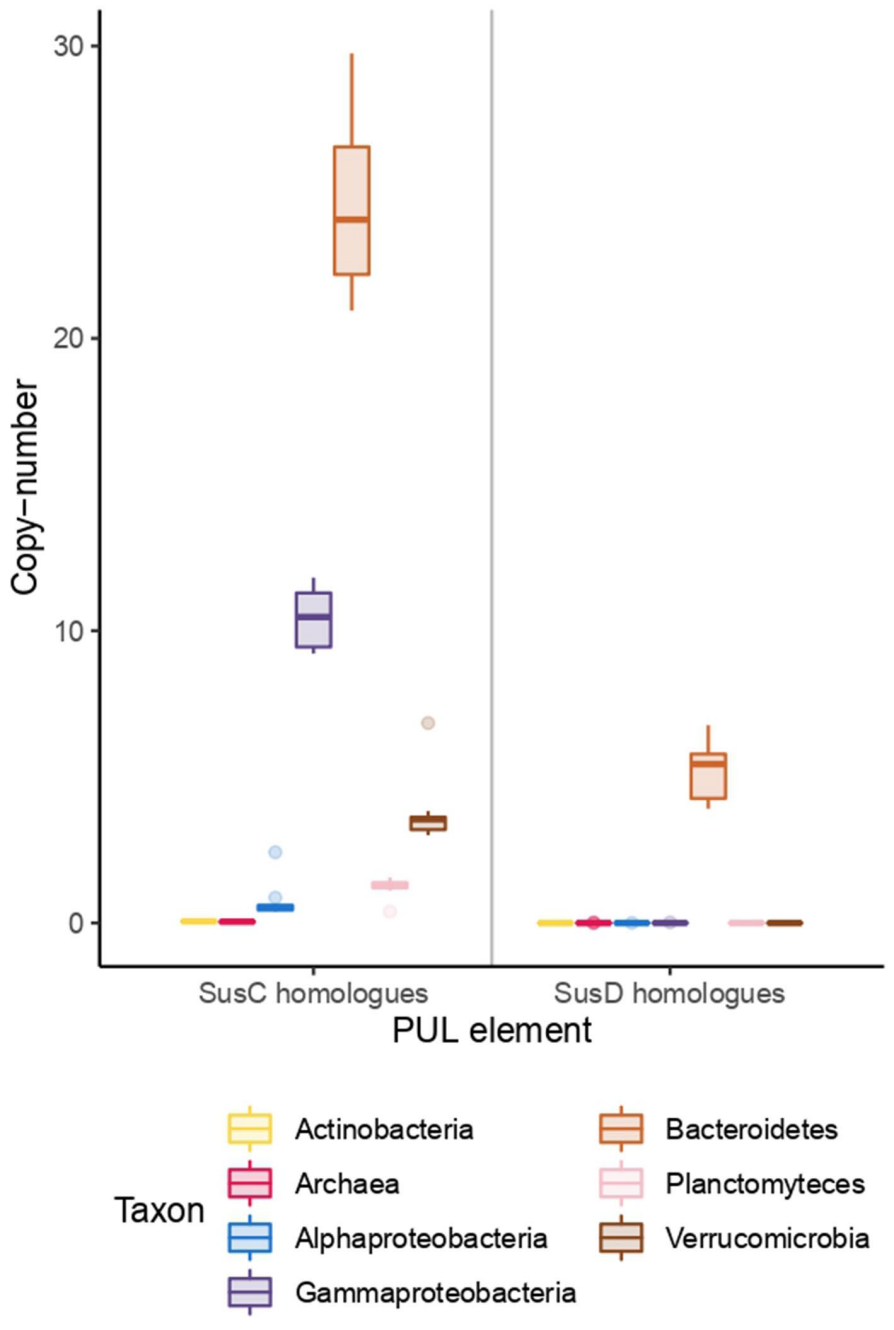
Copy number of SusC and SusD homologs among taxonomic groups. Each box represents copy number variations across the time series, with the middle line representing the median value.

Uptake proteins, namely TonB-dependent transporters (SusC), and associated proteins, such as the polysaccharide-binding protein SusD, also showed a differential distribution among the phyla (Figure 4). SusC transporters were most abundant in Bacteroidetes, and the copy number showed a positive correlation with chlorophyll. There were around 21 copies in early spring, rising to almost 27 in June, with a peak of 29 copies in late June (data not shown). Gammaproteobacteria was the second clade with the highest copies of SusC, more than 10, also with a positive correlation with chlorophyll. SusD binding proteins were exclusive of Bacteroidetes, with an average 6 copies per genome.

**Figure 5:**
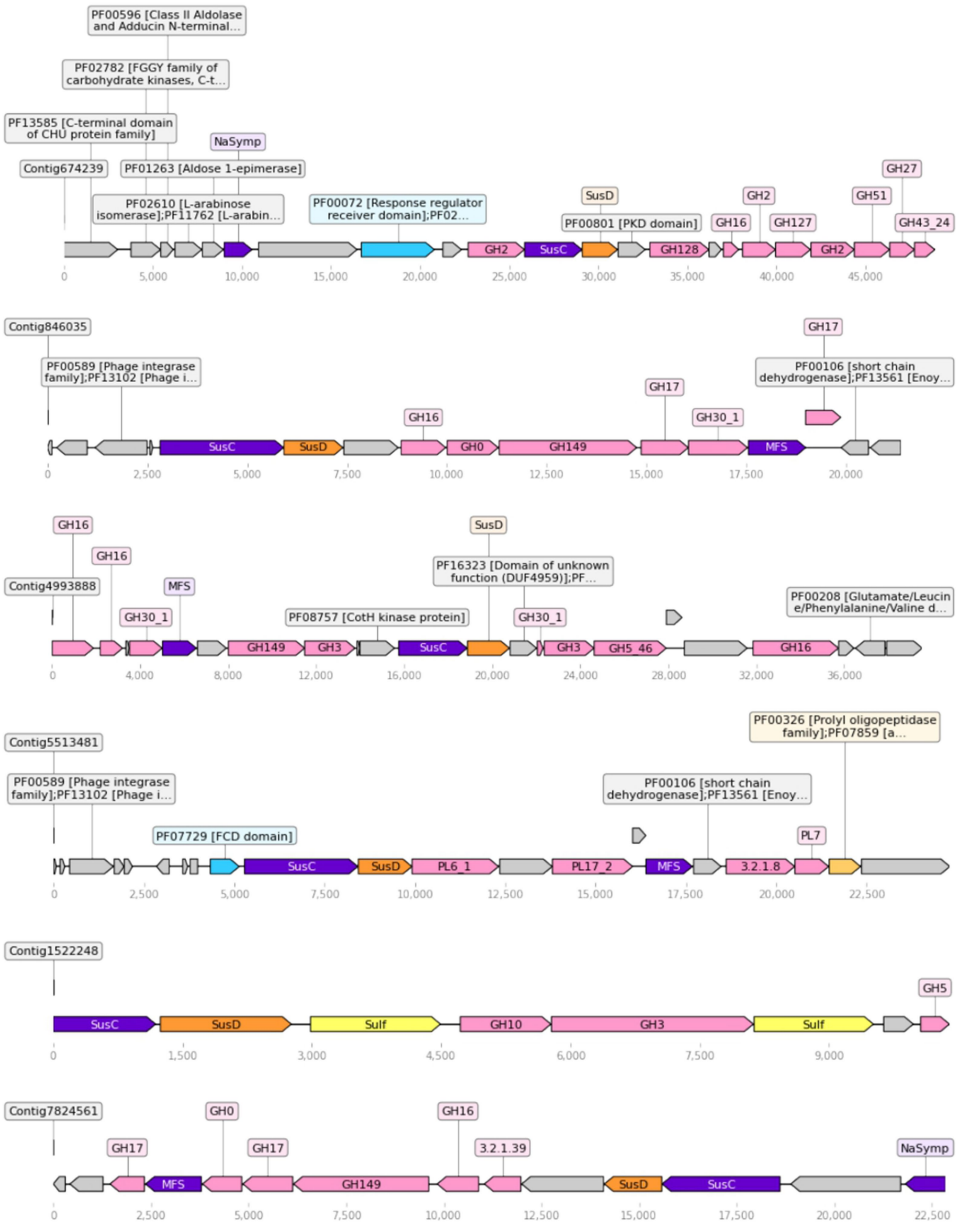
Representation of the biggest PULs found in the dataset. Color key: cazymes (pink), transporters (purple), SusD (orange), sulfatases (yellow), peptidases (gold), regulators (cyan), other (gray). Figure made with dna_feature_viewer.

Four hundred twenty nine unique SusC/D tandems were detected in the dataset. Out of these, 400 were in contigs annotated as Bacteroidetes, 26 were unclassified, 2 were annotated as Proteobacteria and 1 as Marinimicrobia. When adjacent functions were searched to detect a possible PUL around the tandem, about half of the tandems were found to be part of a small contig with only those two genes. On the other hand, 189 tandems were found in contigs big enough to contain at least other three genes, and 61 had at least three cazymes in the contig. Out of these, 34 could be called PULs, as they had three cazymes in proximity and had all nearby cazymes in the same direction as the SusC-D tandem. Out of these 34 detected PULs, half could be taxonomically classified to the Flavobacteriaceae family. These candidate PULs usually included additional genes related with sugar metabolism, such as sulfatases or monosaccharide transporters (MFS, Na-symporters). Some also had a regulatory protein, which is thought to regulate the transcription of the PUL (Figure 5). Within these PULs, functions seem to target a single polysaccharide. As an example, one PUL in contig 846,035 was found to contain five enzymes targeting *β*-1,3-glucans, another in contig 5,513,482 had three alginate lyases, and one PUL in contig 674,239 concentrated up to seven enzymes targeting arabinogalactans.

## 4 Discussion

Algal blooms are seasonal events characterized by a steep increase in phytoplankton abundance, a process that fixes tons of carbon from the atmosphere into the biosphere. These blooms trigger the flux of carbon along the trophic chains, with bacterioplankton being the main agent in the remineralization of the fixed carbon. Bacteroidetes are a phylum that consistently stands out as taking the most advantage of the new substrates. These bacteria possess a diverse set of enzymes, known as cazymes, that degrade algal polysaccharides. The current work has extended these studies to Arctic Bacteroidetes, of which little was known.

Within the bacterial community, the polysaccharide degradative capability was distributed unevenly. Bacterial clades significantly diverged in their GH and PL content, but GT and CE profiles were more similar among the studied clades. GT were mostly related with glycoconjugate and bacterial cell wall biosynthesis, not with polysaccharide degradation, which can explain why GT profiles diverged the least. Archaea did not show capability to degrade polysaccharides, and the only functions they contained could be related to glycoprotein synthesis. Proteobacteria had relatively low copy numbers of cazymes, with a higher diversity of functions in Gammaproteobacteria than in Alphaproteobacteria. Verrucomicrobia and Planctomycetes stood out as having up to tenfold as many sulfatases as the other clades. These high numbers are consistent with those obtained in genomic studies of Planctomycetes (Glöckner et al., 2003) and Verrucomicrobia (Sichert et al., 2020) species. These two phyla also had high copy numbers for degradative cazymes (GH, PL, and CE) with similar functional profiles. This may indicate some similarities in the strategy and the carbon sources used by Planctomycetes and Verrucomicrobia, which both form part of the PVC superphylum (Wagner and Horn, 2006).

Verrucomicrobia have been proposed as relevant polysaccharide degraders in Arctic fjords (Cardman et al., 2014), but their low abundance in the Arctic Ocean and the lack of a measurable response to the algal bloom does not suggest such a role in this environment. They have also been reported as potential fucoidan degraders (Sichert et al., 2020), and the presence of GH29 (fucosidase) in their cazyme repertoire supports this. They, together with Placntomycetes, also had a cazyme repertoire with abundant arabinosidases (GH27, GH43), even though arabinose is not one of the main monomers in phytoplankton polysaccharides. These phyla might well be specialized in lesser-known and hydrolysis-resistant substrates or in minor monomers present as decorations in the main polysaccharides, which may account for their lower abundance as they may feed slowly on these polymers. One of these proposed substrates could be a diatom sulfated fucan polysaccharide that was described as resistant to enzymatic degradation in the North Sea (Vidal-Melgosa et al., 2021). Another possible explanation for this disadvantage could be the absence of siderophore transporters in these phyla, which might hinder their growth by reducing their competitiveness for iron uptake (Puente-Sánchez et al., 2022).

Although literature comments on the abundance and diversity of cazymes in Bacteroidetes, and they relate their success with that genetic repertoire, the current work shows that other clades, namely Verrucomicrobia and Planctomycetes, have a similar amount and diversity of cazymes, yet they only constitute a minority in Arctic bacterioplankton and do not grow in response to the algal bloom. This shifts the attention of the amount and variety of cazymes to the use that these bacteria make of such enzymes. As has been reported in other bloom studies, Bacteroidetes was the clade that most rapidly and most intensely grew with the onset of the algal bloom, dominating all the other clades within months. The increase in the abundance of the phylum was related with species that had a higher cazyme content. Those cazymes related to the degradation of laminarin and mannan were especially enriched, showing a positive correlation with chlorophyll. Laminarin is the main storage polysaccharide in diatoms and some studies indicate that diatom cell walls are most likely glucuromannans (Gügi et al., 2015). *β*-1,4-Mannans have also been detected during phytoplankton blooms and determined to be degraded by bacterial communities (Vidal-Melgosa et al., 2021). Thus, the change in Bacteroidetes species shows a clear trend toward species specialized in diatom polysaccharide degradation. The presence of mannan and glucan phosphorylases (GH130, GH149) in this phylum was unexpected, as most marine polysaccharides have sulfate decorations, but no phosphate. A closer look at the CAZy annotation of these families showed that both target oligosaccharides. This may indicate that these enzymes are not secreted to degrade the extracellular polysaccharide, but instead are kept in the intracellular medium or in the periplasm to phosphorylate absorbed oligosaccharides and avoid their diffusion to the extracellular medium.

Bacteroidetes have an exclusive feature that makes them unique among all other clades, the PUL loci. These operons are characterized by two proteins, SusC and SusD. SusD proteins are polysaccharide-binding proteins that, together with SusC transporters, form a complex that binds oligosaccharides and transports them into the periplasm of bacteria. SusD proteins are exclusive to the phylum Bacteroidetes. The abundance and variety of cazymes was not an exclusive feature that could unequivocally explain Bacteroidetes dominance during the bloom. However, these regulated loci can justify the better adaptation of Bacteroidetes to the degradation of algal polysaccharides. The enzymes associated with each SusC/D tandem seemed to be consistent with the idea that each PUL targets a specific polysaccharide (Martens et al., 2008; Martens et al., 2009). The identification of PULs in metagenomic sequences was challenging, as it required the assembly of long contigs to confirm the presence of relevant functions around the SusC/D tandem. This technical problem might be overcome in future studies by complementing the data with long-read sequences. In future studies, more attention should be placed at the expression of these PULs, as their success might precisely come from a fine-tuned regulation that maximizes the benefit obtained by Bacteroidetes when they synthesize the enzymes.

## Data Availability

The datasets presented in this study can be found in online repositories. The names of the repository/repositories and accession number(s) can be found below: https://www.ncbi.nlm.nih.gov/, ID PRJNA803814.

## Footnotes

^1^ https://www.cnag.crg.eu
