# Corrigendum: Specialized Bacteroidetes dominate the Arctic Ocean during marine spring blooms

**DOI:** 10.3389/fmicb.2025.1534826

**Published:** 2025-01-28

**Authors:** Álvaro Redondo-Río, Christopher J. Mundy, Javier Tamames, Carlos Pedrós-Alió

**Affiliations:** ^1^Department of Systems Biology, Centro Nacional de Biotecnología, CSIC, Madrid, Spain; ^2^Centre for Earth Observation Science, Clayton H. Riddell Faculty of Environment, Earth, and Resources, University of Manitoba, Winnipeg, MB, Canada

**Keywords:** metagenomics, marine, Arctic, Bacteroidetes, cazymes, polysaccharides

In the published article, there was an error in the **Funding** statement. The funding grant by MICIU/AEI/10.13039/501100011033 and by FEDER, UE was omitted. The correct Funding statement appears below:

