# Supplementary material for: Specialized Bacteroidetes dominate the Arctic Ocean during marine spring blooms
Source: Front Microbiol. 2024 Nov 5;15:1481702. doi: 10.3389/fmicb.2024.1481702 (PMC11573768; doi:10.3389/fmicb.2024.1481702)
Supplement: Supplementary file 1 [file Table_1.DOCX]

**Supplementary table 1**. Number of reads and mapped reads for each sample.

| **# Sample** | **Total reads** | **Reads mapping to assembly** | **Mapping percentage** |
| --- | --- | --- | --- |
| 9_Mar | 125074094 | 113413500 | 90.7 |
| 13_Mar | 59727336 | 53683468 | 89.9 |
| 17_Mar | 21538100 | 19514486 | 90.6 |
| 23_Apr | 49198506 | 46060030 | 93.6 |
| 1_May | 175932218 | 166272180 | 94.5 |
| 5_May | 138625032 | 126968342 | 91.6 |
| 10_May | 78507842 | 71317694 | 90.8 |
| 19_May | 74655984 | 68633322 | 91.9 |
| 1_Jun | 87152814 | 80861160 | 92.8 |
| 11_Jun | 145220022 | 135669374 | 93.4 |
| 15_Jun | 82445406 | 71402658 | 86.6 |
| 23_Jun | 28946670 | 26775736 | 92.5 |
| 30_Jul | 100424606 | 93968198 | 93.6 |
