# Supplementary material for: Specialized Bacteroidetes dominate the Arctic Ocean during marine spring blooms
Source: Front Microbiol. 2024 Nov 5;15:1481702. doi: 10.3389/fmicb.2024.1481702 (PMC11573768; doi:10.3389/fmicb.2024.1481702)
Supplement: Supplementary file 2 [file Table_2.DOCX]

**Supplementary table 2**. Details of the assembly.

| Number of contigs | 8166358 |
| --- | --- |
| Total length | 5104467821 |
| Longest contig | 725344 |
| Shortest contig | 200 |
| N50 | 790 |
| N90 | 281 |
| Contigs at superkingdom (k) rank | 6015636 (73.7%), in 4 superkingdoms |
| Contigs at phylum (p) rank | 5330205 (65.3%), in 187 phyla |
| Contigs at class (c) rank | 4210815 (51.6%), in 203 classes |
| Contigs at order (o) rank | 3165768 (38.8%), in 430 orders |
| Contigs at family (f) rank | 2580979 (31.6%), in 696 families |
| Contigs at genus (g) rank | 1413733 (17.3%), in 1654 genera |
| Contigs at species (s) rank | 900102 (11.0%), in 1408 species |
| Number of ORFs | 11057885 |
| Number of rRNAs | 7349 |
| Number of tRNAs/tmRNAs | 50457 |
| ORFs by Aragorn | 50457 |
| ORFs by Prodigal | 10826824 |
| ORFs by barrnap | 7349 |
| ORFs by blastx | 173252 |
| Orphans (no hits) | 2713540 |
| No tax assigned (with hits) | 292959 |
| KEGG annotations | 5380279 |
| COG annotations | 5878147 |
| Pfam annotations | 3349725 |
| CAZy annotations | 537366 |
