# Supplementary material for: Specialized Bacteroidetes dominate the Arctic Ocean during marine spring blooms
Source: Front Microbiol. 2024 Nov 5;15:1481702. doi: 10.3389/fmicb.2024.1481702 (PMC11573768; doi:10.3389/fmicb.2024.1481702)
Supplement: Supplementary file 3 [file Table_3.DOCX]

**Supplementary table 3.** Universal Single Copy Genes (USiCGs) used for the calculation of copy number and the annotations by which they were retrieved.

| **Gene** | **PFAM annotation** |
| --- | --- |
| RplA | PF00687 [Ribosomal protein L1p/L10e family] |
| RplM | PF00572 [Ribosomal protein L13] |
| RplN | PF00238 [Ribosomal protein L14p/L23e] |
| RplO | PF00828 [Ribosomal proteins 50S-L15, 50S-L18e, 60S-L27A] |
| RplF | PF00347 [Ribosomal protein L6] |
| RpsJ | PF00338 [Ribosomal protein S10p/S20e] |
| RpsK | PF00411 [Ribosomal protein S11] |
| RpsL | PF00164 [Ribosomal protein S12/S23] |
| RpsM | PF00416 [Ribosomal protein S13/S18] |
| RpsG | PF00177 [Ribosomal protein S7p/S5e] |
| RpsH | PF00410 [Ribosomal protein S8] |
