# Supplementary material for: Specialized Bacteroidetes dominate the Arctic Ocean during marine spring blooms
Source: Front Microbiol. 2024 Nov 5;15:1481702. doi: 10.3389/fmicb.2024.1481702 (PMC11573768; doi:10.3389/fmicb.2024.1481702)
Supplement: Supplementary file 4 [file Table_4.DOCX]

**Supplementary Table 4.** Genomes from Bacteroidetes species used as a reference to test the performance of the consensus annotation filters.

| **Patric ID** | **Species** |
| --- | --- |
| 1347342.6 | Formosa agariphila KMM 3901 |
| 313598.6 | Polaribacter sp. MED152 |
| 313590.8 | Dokdonia sp. MED134 |
| 1798225.3 | Formosa sp. Hel1_31_208 |
| 2058137.3 | Polaribacter sp. ALD11 |
| 1336795.4 | Formosa sp. Hel3_A1_48 |
| 1336804.3 | Polaribacter sp. Hel1_33_78 |
| 1336794.4 | Formosa sp. Hel1_33_131 |
| 376686.1 | Flavobacterium johnsoniae UW101 |
