# Supplementary material for: Specialized Bacteroidetes dominate the Arctic Ocean during marine spring blooms
Source: Front Microbiol. 2024 Nov 5;15:1481702. doi: 10.3389/fmicb.2024.1481702 (PMC11573768; doi:10.3389/fmicb.2024.1481702)
Supplement: Supplementary file 5 [file Table_5.DOCX]

**Supplementary table 5.** Chi-squared distance values for the four degradative CAZy families (GH, GT, PL and CE) for the seven main taxa.

| **GH** | **Verru.** | **Plancto.** | **Archaea** | **Actino.** | **Alpha.** | **Gamma.** |
| --- | --- | --- | --- | --- | --- | --- |
| **Plancto.** | 1.361 |  |  |  |  |  |
| **Archaea** | 2.34 | 2.431 |  |  |  |  |
| **Actino.** | 1.956 | 2.13 | 2.144 |  |  |  |
| **Alpha.** | 2.256 | 2.408 | 2.596 | 2.14 |  |  |
| **Gamma.** | 1.91 | 2.127 | 2.148 | 1.719 | 1.416 |  |
| **Bacteroidetes** | 1.738 | 1.993 | 2.127 | 1.917 | 2.007 | 1.625 |
| **GT** |  |  |  |  |  |  |
| **Plancto.** | 0.687 |  |  |  |  |  |
| **Archaea** | 1.473 | 1.29 |  |  |  |  |
| **Actino.** | 0.873 | 0.805 | 1.269 |  |  |  |
| **Alpha.** | 0.99 | 1.037 | 1.65 | 1.131 |  |  |
| **Gamma.** | 1.119 | 1.169 | 1.73 | 1.176 | 0.906 |  |
| **Bacteroidetes** | 0.671 | 0.593 | 1.288 | 0.696 | 0.908 | 1.041 |
| **PL** |  |  |  |  |  |  |
| **Plancto.** | 2.238 |  |  |  |  |  |
| **Archaea** | 1.719 | 2.317 |  |  |  |  |
| **Actino.** | 2.603 | 2.687 | 2.433 |  |  |  |
| **Alpha.** | 1.95 | 2.529 | 0.97 | 2.697 |  |  |
| **Gamma.** | 2.192 | 2.407 | 1.903 | 2.45 | 2.025 |  |
| **Bacteroidetes** | 2.265 | 2.645 | 2.355 | 2.876 | 2.468 | 1.821 |
| **CE** |  |  |  |  |  |  |
| **Plancto.** | 0.871 |  |  |  |  |  |
| **Archaea** | 1.466 | 1.53 |  |  |  |  |
| **Actino.** | 1.612 | 1.49 | 1.127 |  |  |  |
| **Alpha.** | 1.544 | 1.726 | 1.374 | 1.795 |  |  |
| **Gamma.** | 1.27 | 1.461 | 1.223 | 1.671 | 0.745 |  |
| **Bacteroidetes** | 1.347 | 1.345 | 0.862 | 1.295 | 1.364 | 1.031 |
