# Supplementary material for: Specialized Bacteroidetes dominate the Arctic Ocean during marine spring blooms
Source: Front Microbiol. 2024 Nov 5;15:1481702. doi: 10.3389/fmicb.2024.1481702 (PMC11573768; doi:10.3389/fmicb.2024.1481702)

Supplementary figure 1. Glycosyl hydrolase (GH) profiles of different taxa, showing the percentage that each family represents out of the total copy number. GH families whose abundance was under the 60% quartile were grouped under the "Minor" tag to ease visualization.

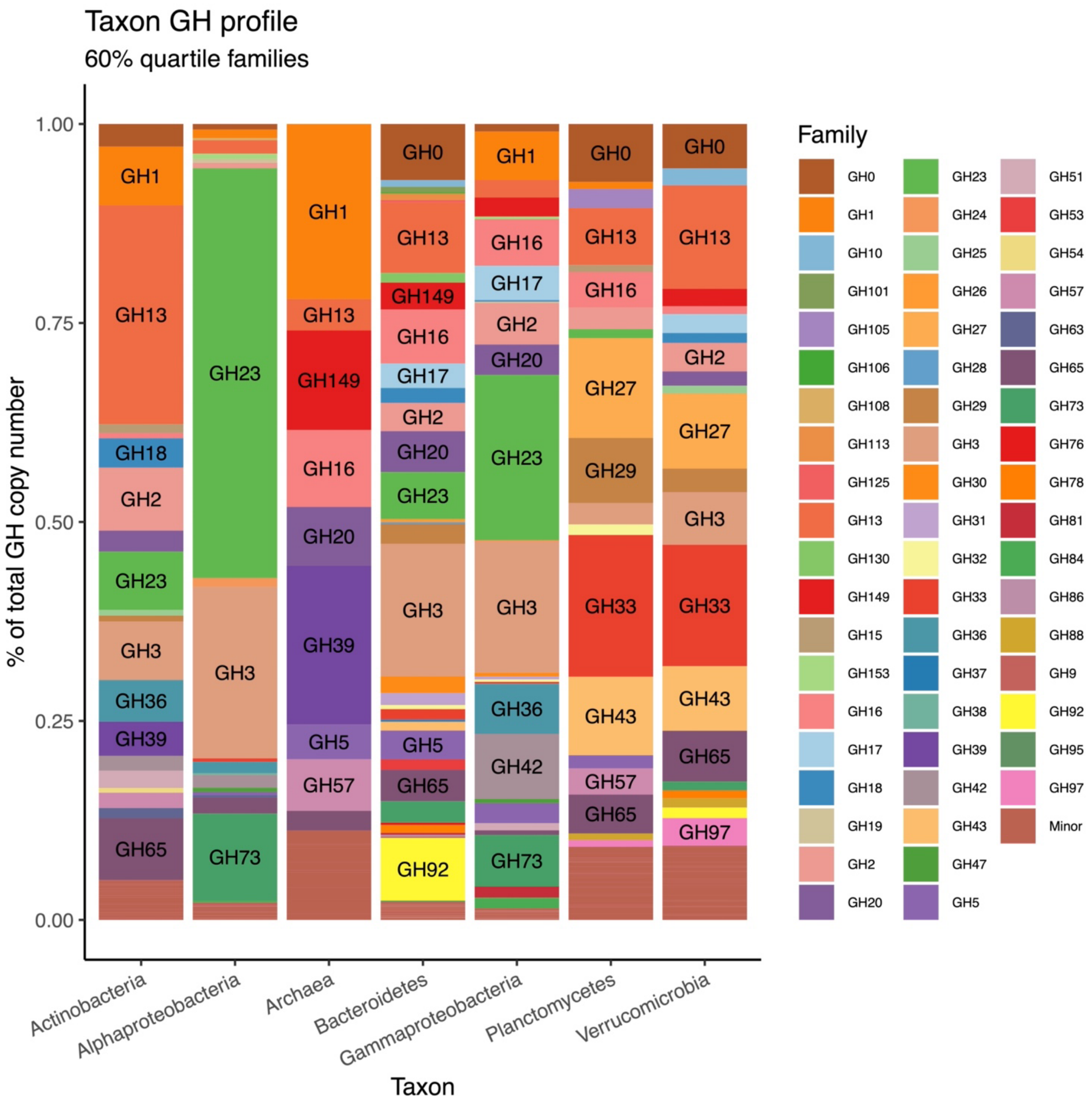

Supplement: Supplementary file 6 [file Image_1.PDF]
