# Supplementary material for: Specialized Bacteroidetes dominate the Arctic Ocean during marine spring blooms
Source: Front Microbiol. 2024 Nov 5;15:1481702. doi: 10.3389/fmicb.2024.1481702 (PMC11573768; doi:10.3389/fmicb.2024.1481702)

Supplementary figure 4. Carboxyl esterase (CE) profiles of different taxa, showing the percentage that each family represents out of the total copy number.

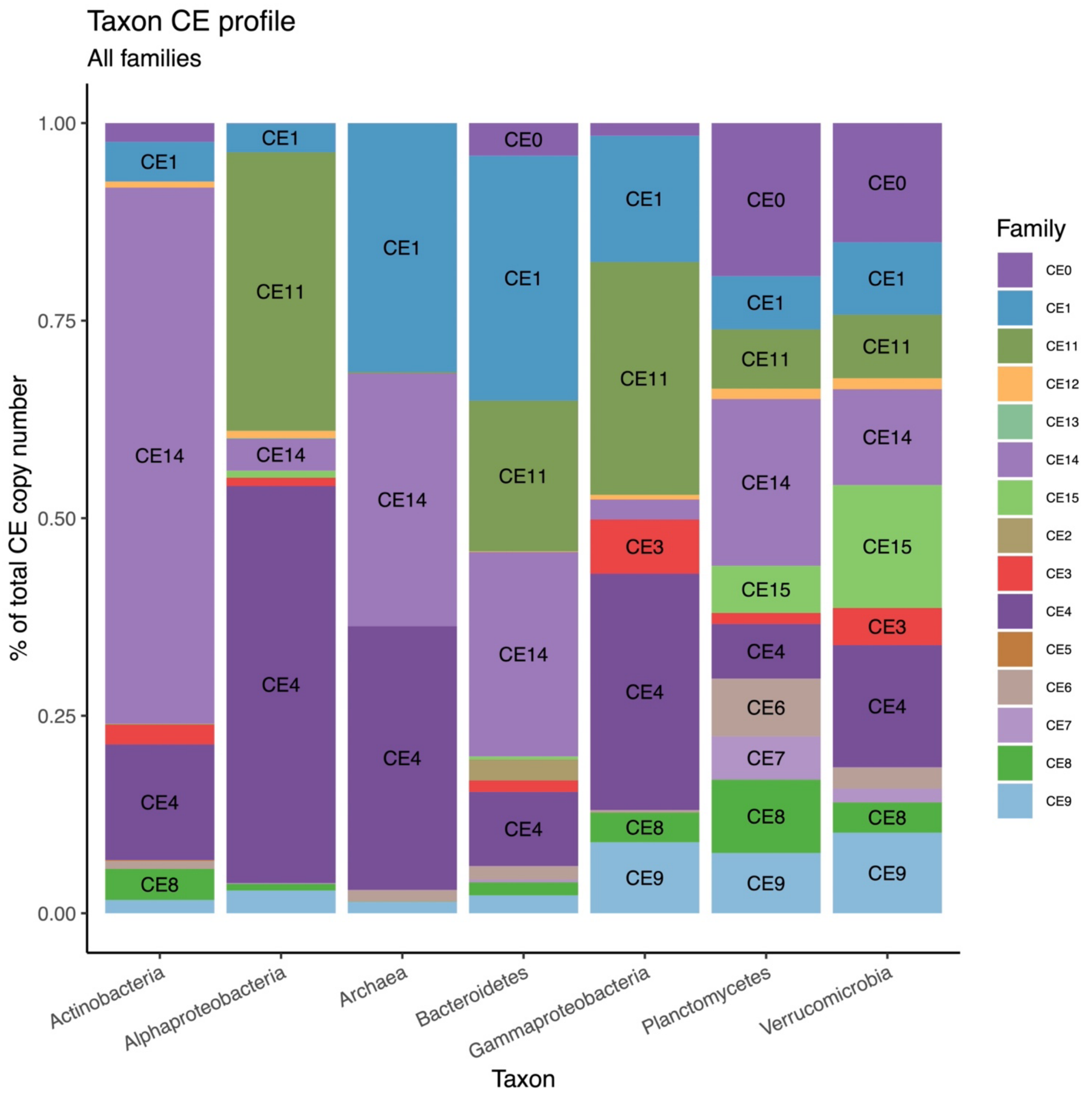

Supplement: Supplementary file 9 [file Image_4.PDF]
