# Supplementary material for: Specialized Bacteroidetes dominate the Arctic Ocean during marine spring blooms
Source: Front Microbiol. 2024 Nov 5;15:1481702. doi: 10.3389/fmicb.2024.1481702 (PMC11573768; doi:10.3389/fmicb.2024.1481702)

Supplementary figure 5. Heatmap showing the copy number of GH families (trcn, log2 scale) across the time series. Only families showing a significant (corrected p-val ≤ 0.01) correlation with chlorophyll A are shown. Families are ordered by their correlation index, positive (bottom) to negative (top).

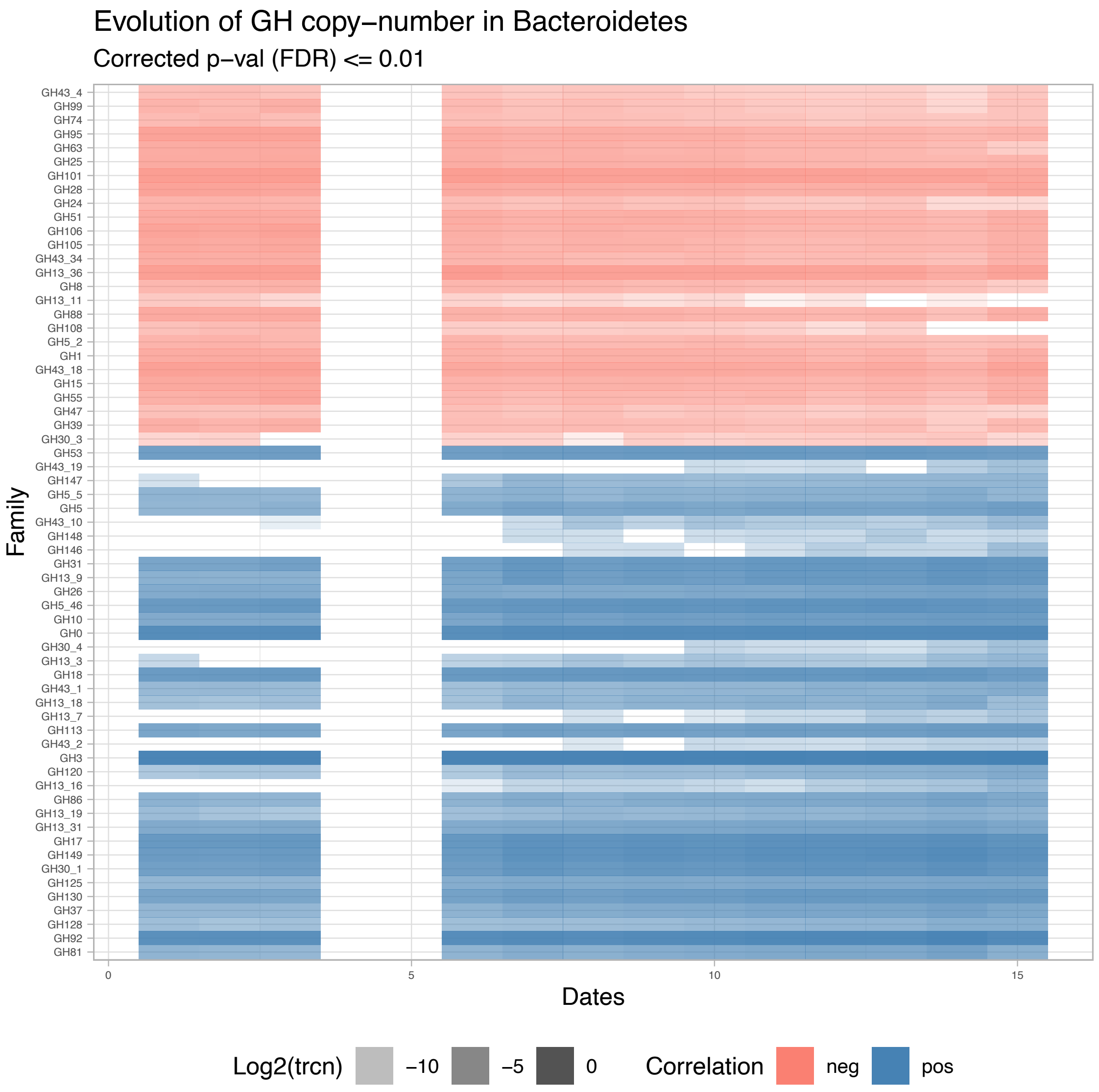

Supplement: Supplementary file 10 [file Image_5.PDF]
